# Placental Mesenchymal Dysplasia With Normal Fetus: A Rare Case Report

**Published:** 2017-07-01

**Authors:** Subrata Pal, Kingshuk Bose, Prabhat Ch Mondal, Srabani Chakrabarti, Mrinal Sikder

**Affiliations:** 1 *Dept. of Pathology, College of Medicine and Sagore Dutta Hospital. Kolkata, India*; 2 *Dept. of Pathology, Bankura Sammilani Medical College, Bankura, India. *; 3 *Dept. of Gynaecology and Obstretics, Bankura Sammilani Medical College, Bankura, India*; 4 *Dept. of Pathology, Culcutta National Medical College, Kolkata, India*; 5 *Dept. of Pathology, R G Kar Medical College, Kolkata, India*

**Keywords:** Placental Mesenchymal, Dysplasia Maxilla, Normal Fetus, Partial Mole

## Abstract

Placental mesenchymal dysplasia (PMD) is a rare benign placental abnormality. It is characterized by hydropic degeneration of stem villi, placentomegaly, and increased maternal serum alpha-fetoprotein (AFP). It can be associated with different congenital abnormalities, karyotype abnormalities, and feto-maternal morbidities. It is difficult to differentiate PMDfrom partial mole, complete mole with twin pregnancy in ultrasound, and in macroscopic examination. The current paper presentsa rare case of placental mesenchymal dysplasia in a young primigravida mother who delivered a normal fetus withnormal karyotype.

## Introduction

Placental mesenchymal dysplasia (PMD) is a rare benign condition characterized by placentomegalyand grape-like vesicles (1,2).The incidence of PMD is 0.02% of pregnancy cases and it commonly affects the female fetus (F:M 4:1)(2,3). Pathologically, it is a benign disorder of stem villi with elevated serum alpha-fetoprotein (AFP)(1,2). Majority of the cases are associated with intrauterine growth restriction (IUGR), intrauterine fetal death (IUFD), and rarely with various congenital abnormalities such asmacrosomia, exomphalos, omphalocele, visceromegaly, and macroglossia(2,4). But, PMD cases may be associated with normal fetus (2,4). In ultrasound and gross examinations, PMD mimics partial mole, complete mole with twin pregnancy,and chorioangioma with normal fetus (1).The current paper presents a rare case of PMD with a normal fetus.

## Case Report

**Figure 1:**
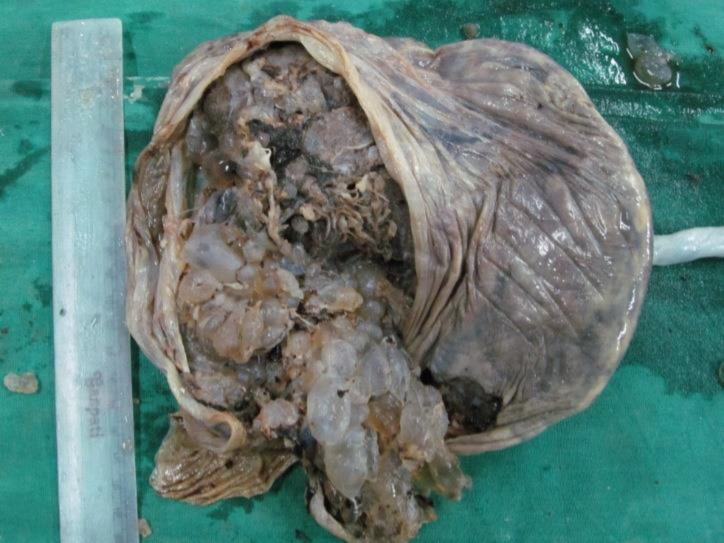
Gross image of enlarged placenta with multiple grape-like vesicles at maternal surface

A 22-year-old primigravida mother from tribal region was admitted in antenatal ward with labor pain at the 37th week of pregnancy. She was under the clinical checkup of rural health center during the pregnancy. She was immunized with tetanus toxoid and iron supplement was also given. The casewas normotensive and had an average built. All the hematological tests were normal, except mild anemia (hemoglobin 10 g%). Ultrasound evaluation revealed a single viable fetus and multiple hypoechoic cystic structures without vascular flow. Radiological features were suggestive of partial mole with a live intrauterine fetus. Serum AFP and β-hCGlevelswere 485ng/mL and 25780mIU/L, respectively. She underwent normal delivery at the 37th week of pregnancy. She gave birth to a female baby with 2450g birth weight. Apgar score of the baby was eightand nineat the minutesone and five. General examination of the baby at birth did not reveal any congenital abnormality. The placenta was large and bulky (20x20x3cm); weighed 950g. The umbilical cord was 56 cm long and 1cm indiameter. The cord was eccentrically inserted at placenta. The placenta had plenty of grape-like vesicles also with some normal appearing villous tissue (Figure 1). The placenta andcord weresent for histopathological examinations. 

**Figure 2:**
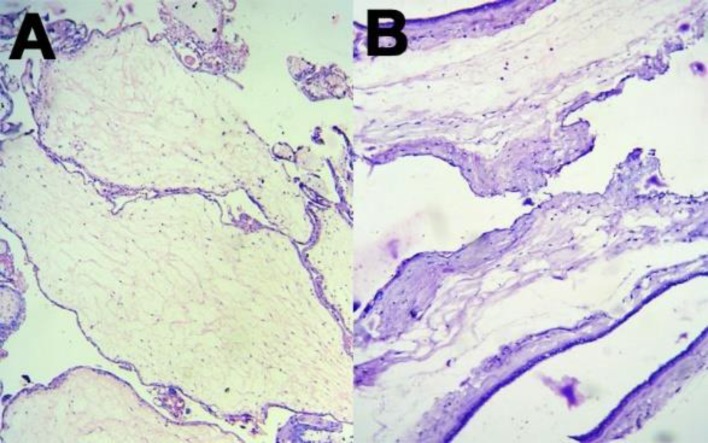
Photomicrograph showing placental mesenchymal dysplasia,dilated stem villi with cistern formation (A) and trophoblast lining (B) (H & E stain, low-power view)

**Figure 3:**
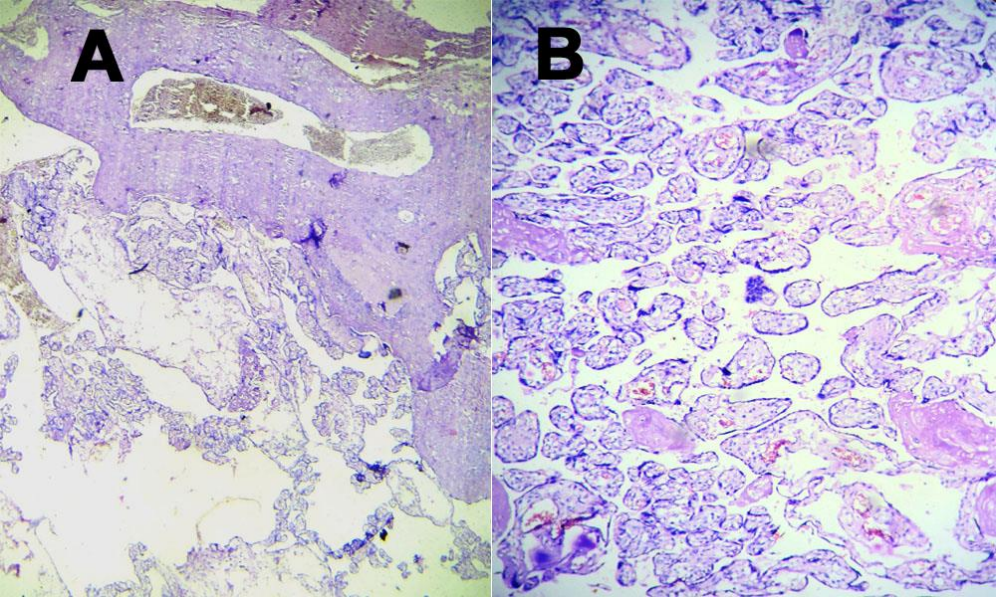
Photomicrograph showing fibromuscular stem villi (A) with normal terminal villi, (B) without trophoblastic proliferation (H &E stain, scanner and low-power view)

On histopathology, the sections revealed edematous stem villi with cisterns formation (Figure 2). The blood vessels were dilated and the walls were thickened with the presence of fibromuscular hyperplasia (Figure 3A). Trophoblastic proliferation was absent and terminal villi were normal (Figure 3B). The histology of umbilical cord was normal. PMD was diagnosed on histopathology. Karyotyping of the baby was 46XX without any other abnormalities. 

.

## Discussion

PMD is a rare placental vascular abnormality (1,2). The term was first introducedby Mosooso et al., in 1991 (4,5). The entity is characterized by enlarged placenta, cystic dilatation of stem villi with the formation of grape-like vesicles and dilated vasculatures. The incidence of PMD was0.02% in the literatures (6). PMD cases werefrequently associated with the Beckwith-Wiedemannsyndrome (23%), IUGR (33%), IUFD (13%),and preterm labor (33%)(6,7). 

The etiology of PMD is not clear. It is postulated that different heterogeneous factors such asandrogenic or biparentalmosaicism, congenital mesodermal malformation, hypoxia, and hypoperfusion of unknown etiology stimulate fibroblastic proliferation (4-6,8). Increased fibroblast proliferation causes excessive production of vascular endothelial growth factor(VEGF), which stimulates angiogenesis and vascular malformation. Most of the cases have normal karyotype (89%), but it may be associated with the Beckwith-Wiedemannsyndrome (23%), trisomy (13%), the Klinefeltersyndrome, 69XXY, or triploidysyndrome (5,9,10).

Most of the PMD cases have no definite clinical manifestation. The cases are incidentally diagnosed during the prenatal ultrasound examination for routine checkup (2).Atultrasound examination, PMD shows hypoechoic thickened placenta and it is often indistinguishable from partial mole, complete mole with co-twin, and chorioangioma associated with normal fetus (1,10). In the present case, ultrasound features were suggestive of partial mole. Laboratory tests of PMD cases reveal increased maternal serum AFP and variable β-hCGlevels (1,5). In thecurrent case, AFP was elevated and β-hCGwas mildly elevated in antenatal period, but fellto normal value on postpartum period. 

On gross examination, PMD shows placentomegalywith large edematous cystic villi admixed with normal terminal villi. Blood vessels of fetal surface show aneurysmal dilatation (1). Sometimes, it is associated with various umbilical cord abnormalities such asexcessive long cord, tortuous twisted cord, abnormal insertion of cord, and single umbilical artery. In the currentcase, the cord was eccentrically inserted. Even in gross examination, it was indistinguishable from partial mole.Microscopy of PMD shows markedly enlarged edematous stem villi and cisterns formation without trophoblastic proliferation (1,2,5). The vessel walls are thickened with fibromuscular hyperplasia, but terminal villi show normal architecture (2,5). In the currentcase, histomorphology was a classic case of PMD. The edematous stem villi stainwas positive with alcian blue, but negative with smooth muscle actin (SMA); where normal villi give positive staining with SMA (4). Immunohistochemistry with desmin and vimentin give positive staining both in PMD and normal villi (4).

Important differential diagnoses of PMD in ultrasound and gross examinations are partial mole, complete mole with twin gestation, multiple chorioangioma with normal fetus, and confined placental mosaicism (2,9). Absence of trophoblastic proliferation and normal diploid karyotype distinguish the case from partial mole (2). In contrast to PMD, complete mole has hydropic degeneration of terminal villi and trophoblastic proliferation (4). Placental mosaicismis excluded by evidence of diploid karyotype of the present case.

## Conclusion

PMD is a rare abnormality and should be differentiated from molar pregnancy, because it does not need termination of pregnancy. It is vital to differentiate macroscopy, microscopy, and karyotyping from other differential diagnoses. In antenatal diagnosis, patients should be counseled about increased feto-maternal morbidity and close follow-up is necessary in antenatal and perinatal periods. 
